# Improving oral health communication in paediatric dentistry using intraoral scanners: a multicentre pilot study

**DOI:** 10.1007/s40368-026-01212-z

**Published:** 2026-04-29

**Authors:** S. Schick, M. A. Schlenz, N. Schulz-Weidner, C. Frese, A. Pelkonen, T. Tanner

**Affiliations:** 1 Clinic for Oral, Dental and Maxillofacial Diseases, Department of Conservative Dentistry, Heidelberg University, Heidelberg, Germany; 2 University Hospital Schleswig-Holstein, Campus Kiel, Department of Prosthodontics, Kiel University, Kiel, Germany; 3 Dental Clinic - Department of Paediatric Dentistry, University of Giessen, Giessen, Germany; 4 Cariology, Endodontology and Paediatric Dentistry, Research Unit of Population Health, Oulu, Finland and The Wellbeing Services County of North Ostrobothnia, Pohde, University of Oulu, Oulu, Finland

**Keywords:** Intraoral scanners, Digital dentistry, Clinical study, Preventive dentistry, Paediatric dentistry, Oral health

## Abstract

**Purpose:**

This multicentre pilot study examined whether intraoral scanners (IOS) enhance oral health competence in paediatric patients and their caregivers compared with conventional verbal oral health instructions (OHI).

**Methods:**

Sixty children aged 6–14 years and their caregivers from three European university centres were randomly assigned to a control group (verbal OHI) or an intervention group (verbal OHI + IOS visualisation). Children were stratified into age groups: (6–8, 9–11, 12–14 years). Calibrated dentists performed standardised examinations and delivered OHI. In the intervention arm, an IOS scan was obtained and the 3D model was used to explain individual clinical findings. Comprehension of clinical findings was assessed after the consultation using age-adapted questionnaires. Children in the intervention group additionally rated their IOS experience using a visual analogue scale (VAS, 0–100%). Statistical analysis included descriptive statistics and Kruskal–Wallis tests.

**Results:**

Children in the intervention group achieved higher comprehension scores (4.40 ± 1.61 vs. 3.33 ± 1.81; *p* = 0.019). Caregivers also scored higher in the intervention group (5.03 ± 1.25 vs. 4.10 ± 1.37; *p* = 0.008). Benefits were greatest in children aged 6–11 years. VAS ratings indicated acceptance: scanning was perceived as “fun” (83.8%), “informative” (81.3%) and “enjoyable to watch” (88.6%). Tip size was rated appropriate (68.7%), whilst agreement with “boring” (25.9%) and “painful” (24.1%) was low. The dentist’s explanation was rated as clear (94.8%).

**Conclusion:**

IOS improved communication and understanding of oral health findings in paediatric dentistry, particularly amongst younger children and their caregivers.

**Supplementary Information:**

The online version contains supplementary material available at https://doi.org/10.1007/s40368-026-01212-z.

## Introduction

Dental caries is one of the most common chronic diseases worldwide, affecting both the primary and permanent dentition in all age groups (Bernabé et al. 2016; GBD 2017 Oral Disorders Collaborators 2020). Dental caries is a multifactorial disease resulting from a shift in the microbial balance (dysbiosis), favouring cariogenic bacteria due to various lifestyle and dietary factors (Takahashi & Nyvad 2008). Frequent consumption of sugary products and poor oral hygiene practices are amongst the modifiable risk factors for caries (Tanner et al. 2013; Kumar et al. 2016; Hujoel & Lingström 2017). For children, these behaviours are particularly influenced by their parents or caregivers, as the oral health habits of children often reflect parental attitudes and practices toward dental care (Finlayson et al. 2007; Vanagas et al. 2009).

Given the long-term consequences of untreated caries lesions in children, such as pain, infection and potential impact on growth and quality of life, preventive and early diagnostic approaches in paediatric dentistry are crucial (Kassebaum et al. 2015). Early caries risk diagnosis enables oral health professionals to decide between preventive and minimally invasive interventions and to provide targeted guidance on diet and oral hygiene practices (Lussi 1993). Traditional visual and tactile examinations remain the primary tools for caries lesion diagnosis, often involving dental mirrors, light sources and manual probing (Pitts et al. 2013). However, these conventional methods have limitations when it comes to effectively improving the oral health competence of children and their caregivers, creating understanding and showing them specific treatment needs.

In recent years, intraoral scanners (IOS) have gained attention as versatile tools in digital dentistry, allowing for precise three-dimensional (3D) imaging of the oral cavity (Richert et al. 2017). Originally developed for use in restorative and prosthetic treatments, IOS technology now holds promise for broader applications, including patient education and preventive care. Studies have demonstrated that IOS can be used effectively to document and monitor dental structures over time, including applications in dental erosion and wear assessment (Alaraudanjoki et al. 2017). In the case of caries lesion management, some IOS offer caries lesion detection tools integrated into the hardware and software of the IOS that may support the detection and monitoring of early carious lesions, although current evidence remains limited (Kühnisch et al. 2016; Schlenz et al. 2022; Schulz-Weidner et al. 2024a, b). Despite these promising applications, data on the use of IOS in routine paediatric dental care, especially for caries lesion diagnosis and oral health education, remain limited (Schlenz et al. 2022).

Visual aids are known to improve patient comprehension and engagement in various healthcare settings, and IOS may serve a similar role in dentistry by offering clear, patient-specific visualisations of the teeth and the surrounding tissues. In dentistry, visual aids complement written and verbal information and can bridge comprehension gaps, particularly for patients with low literacy skills (Jafri et al. 2020). For example, studies have shown that using visual aids can improve patient understanding in contexts such as surgical procedures and chronic disease management (Bernhard et al. 2016). In paediatric dentistry, however, effective communication is further complicated by the need to engage not only young patients but also their parents or primary caregivers, who play a critical role in managing children’s oral health. Unlike adult patients, children have limited oral health literacy, making the involvement of primary caregivers essential for understanding and relaying dental information (Horowitz and Kleinman 2008). Results from Schulz-Weidner and colleagues showed that the visualisation of findings using IOS during patient education led to an improved oral understanding of parents and primary caregivers, which might increase the acceptance of treatment consensus (Schulz-Weidner et al. 2024a, b). These facts underline that these new technologies offer promising enhancements to visual and verbal communication that may improve paediatric dental care outcomes.

Traditional dental models and live intraoral camera feeds have been used to explain oral conditions, but they often fall short as they either lack personalisation or display limited views of the mouth. For some patients, especially younger children, viewing real images of their mouths may be overwhelming or even aversive, due to a lack of familiarity or fear of confronting perceived problems, thereby complicating the explanation process (Rozier et al. 2005; Burzynski et al. 2018). Intraoral scanners (IOS) represent a novel, interactive solution, capturing detailed, coloured 3D models of the entire jaw that can be used to visually communicate specific oral conditions, such as caries lesions or plaque accumulation, in a way that is easier for both children and primary caregivers to understand. These detailed 3D scans provide realistic, individualised views that more closely resemble a digital “model” of the patient’s own mouth, enhancing understanding and engagement in oral care.

This multicentre, cross-sectional pilot study aims to explore the efficacy of IOS as a tool for improving patient education and oral hygiene instruction in children aged 6 to 14 years, along with their primary caregivers. The null hypothesis of the study is that there is no statistically significant difference between the efficacy of conventional verbal oral health instructions (OHI) and the efficacy of patient education and oral hygiene instruction supported by IOS. The objective is to determine whether visualisation of dental conditions using IOS improves comprehension of oral hygiene status, treatment needs, and the rationale for preventive measures such as caries prevention and plaque control.

## Materials and methods

This multicentre, cross-sectional pilot study with random allocation was conducted at three study centres in Europe (two in Germany and one in Finland). The study was approved by the ethical committees of all participating study centres (S-629/2023; ref. no. 46/20; 34/2023).

Participants were recruited from regular dental visits. Children were eligible for inclusion if they were between 6 and 14 years of age and accompanied by a parent or legal primary caregiver, presented with inadequate oral hygiene, and showed a clinical indication for dental treatment (e.g. initial carious lesions, gingivitis, defective restorations). Before randomisation, children were asked whether they had previous experience with intraoral scanners. To avoid bias due to prior familiarity with the technology, those who had already been scanned were not included in the intervention group. Exclusion criteria, therefore, included prior experience with intraoral scanning, severe systemic illness, non-fluency in the local language (German or Finnish) or refusal of informed consent.

Participants were allocated using computer-generated block randomisation lists, stratified by study site. Group allocation was carried out by an independent researcher not involved in recruitment or data collection. At each study centre, ten child/caregiver dyads were randomly assigned to the control group (solely verbal OHI) and ten dyads to the intervention group (verbal OHI and IOS visualisation). In total, 120 participants were enrolled: 60 children and 60 caregivers, forming 60 dyads, evenly allocated to intervention (*n* = 30 dyads) and control (*n* = 30 dyads).

### Age stratification (rationale)

Children were stratified into three age groups (6 to 8 years, 9 to 11 years and 12 to 14 years) to reflect relevant developmental and clinical stages. These bands map onto (a) dentition phases (early/ late mixed dentition, early permanent dentition), (b) age-related differences in cognitive processing (transition from concrete- to formal-operational thinking around early adolescence) and (c) child-specific health literacy considerations, where younger children benefit disproportionately from concrete, visual information and scaffolded communication (Bröder et al. 2017).

To ensure balanced subgroup analysis by age, a stratified randomisation was conducted: each study centre received a predefined number of participants to recruit per age group (6 to 8 years, 9 to 11 years and 12 to 14 years) and group allocation. This ensured an even distribution across the age groups whilst maintaining random assignment at the participant level.

### Intraoral scanner (IOS) and clinical calibration

The intraoral scanning for the intervention group was performed using the IOS Trios 4 (3Shape, Copenhagen, Denmark). The latest software version available at the start of the study was used. Researchers received structured training on the use of IOS Trios 4 prior to the study start. The training covered scanning protocols, anatomical landmarks, and image interpretation relevant to paediatric patients. Scanning was performed according to manufacturer’s instructions. Any uncertainty in interpreting findings was discussed amongst the team to ensure consistent documentation. The training and calibration of researchers were conducted in September 2023.

### Study procedure (within-visit sequence)

At each site, clinical examinations were carried out by calibrated dentists following a shared protocol agreed during the joint calibration session (September 2023). Each child received a standard clinical examination according to World Health Organisation (WHO) criteria, using a dental unit, light, mirror, and probe. Clinical findings (e.g. gingivitis, initial carious lesions, defective restorations) were visually assessed and documented as part of routine care.

Following the examination, children and their caregivers in both groups (control and intervention groups) received targeted verbal OHI about the patients’ oral health status, including details on residual plaque, gingivitis, caries lesions, teeth to be extracted, etc.

In the intervention group, children had their dentition scanned with the Trios 4 after the clinical examination. No caries lesion detection aids or software overlays provided by the manufacturer were used. They then received targeted OHI about the patients’ oral health status using the scan images (Fig. S1).

In both arms, the examining dentist provided the verbal oral hygiene instructions (OHI). In the intervention arm, the same calibrated operator obtained an intraoral scan and used the resulting 3D model to visualise the child’s findings during the explanation.

Children and caregivers had the opportunity to ask clarifying questions. Subsequently, both the child and their caregiver completed a questionnaire assessing their knowledge, responding in their native language (Finnish or German). Additionally, the children in the intervention group were asked about their experience with intraoral scanning. The questionnaire was administered by trained study staff who were not involved in the clinical examination or IOS operation after the child and their caregiver received either OHI or OHI + IOS. Children and caregivers completed their forms separately and independently (no discussion or mutual assistance permitted). For younger children, items could be read aloud verbatim by study staff upon request and caregivers were instructed not to assist the child’s responses.

### Questionnaire

The questionnaires were adapted and expanded based on previously validated instruments by Burzynsky and colleagues (Burzynski et al. 2018) and Schulz-Weidner and colleagues (Schulz-Weidner et al. 2024a, b). To ensure face validity and age-appropriate comprehension, the questionnaires were checked by specialised dentists. In addition, the questionnaires were pilot-tested with a small group of children using the technique of thinking aloud to identify ambiguities and assess clarity. Feedback from both dentists and children was used to finalise the items. The final structure of the questionnaires was approved by the authors SS, MS and TT.

With special regard to the dentition transition status, the dentition images used in the questionnaire accounted for the average status of different age groups, with younger children (6–11 years) having mixed dentition in the model images and older children (12–14 years) having permanent dentition. The questionnaires were paper-based and were answered with a pencil and were completed independently by children and caregivers

**Fig. 1:**
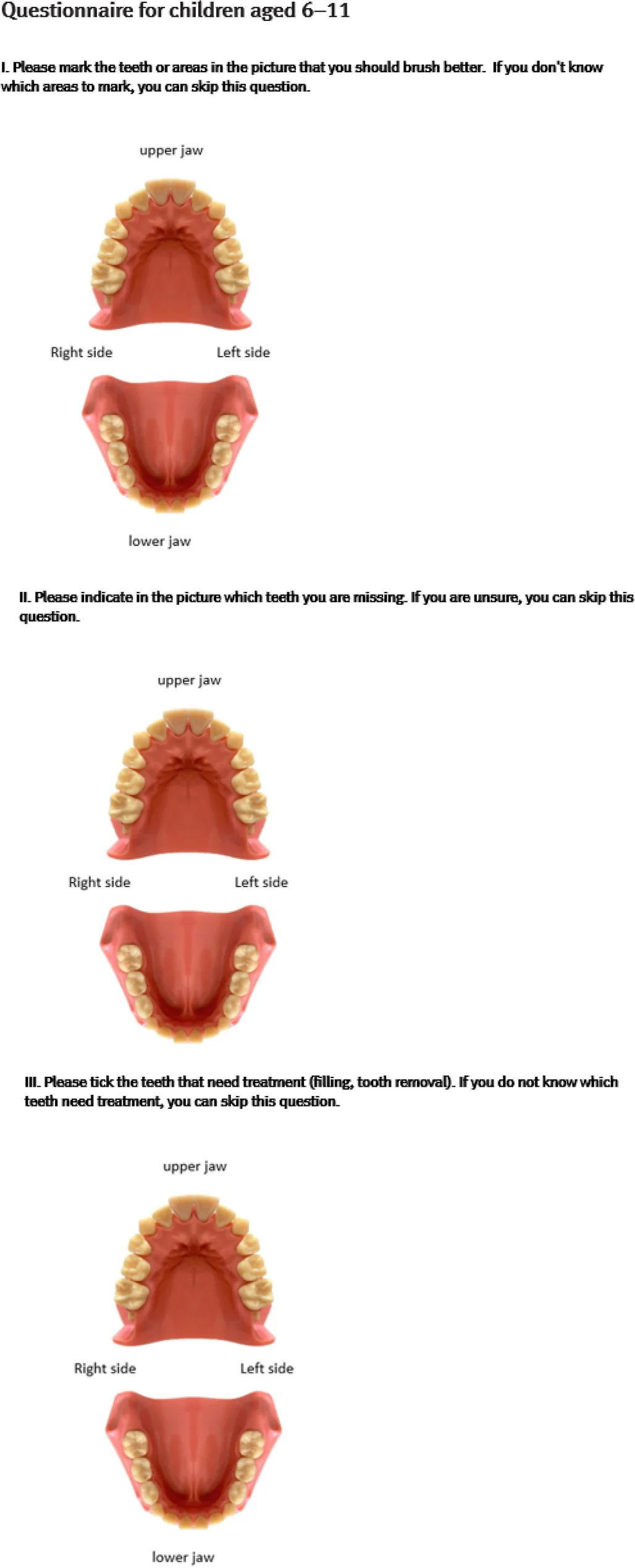
Questionnaire for children (aged 6–11)

**Fig. 2:**
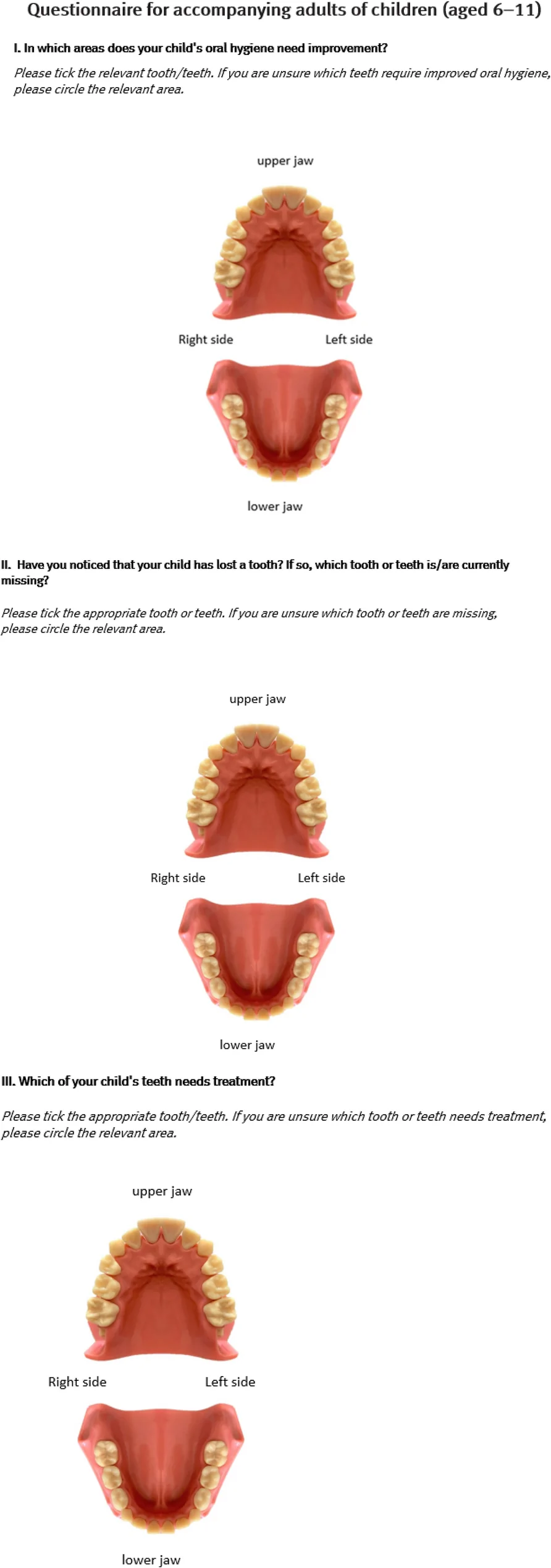

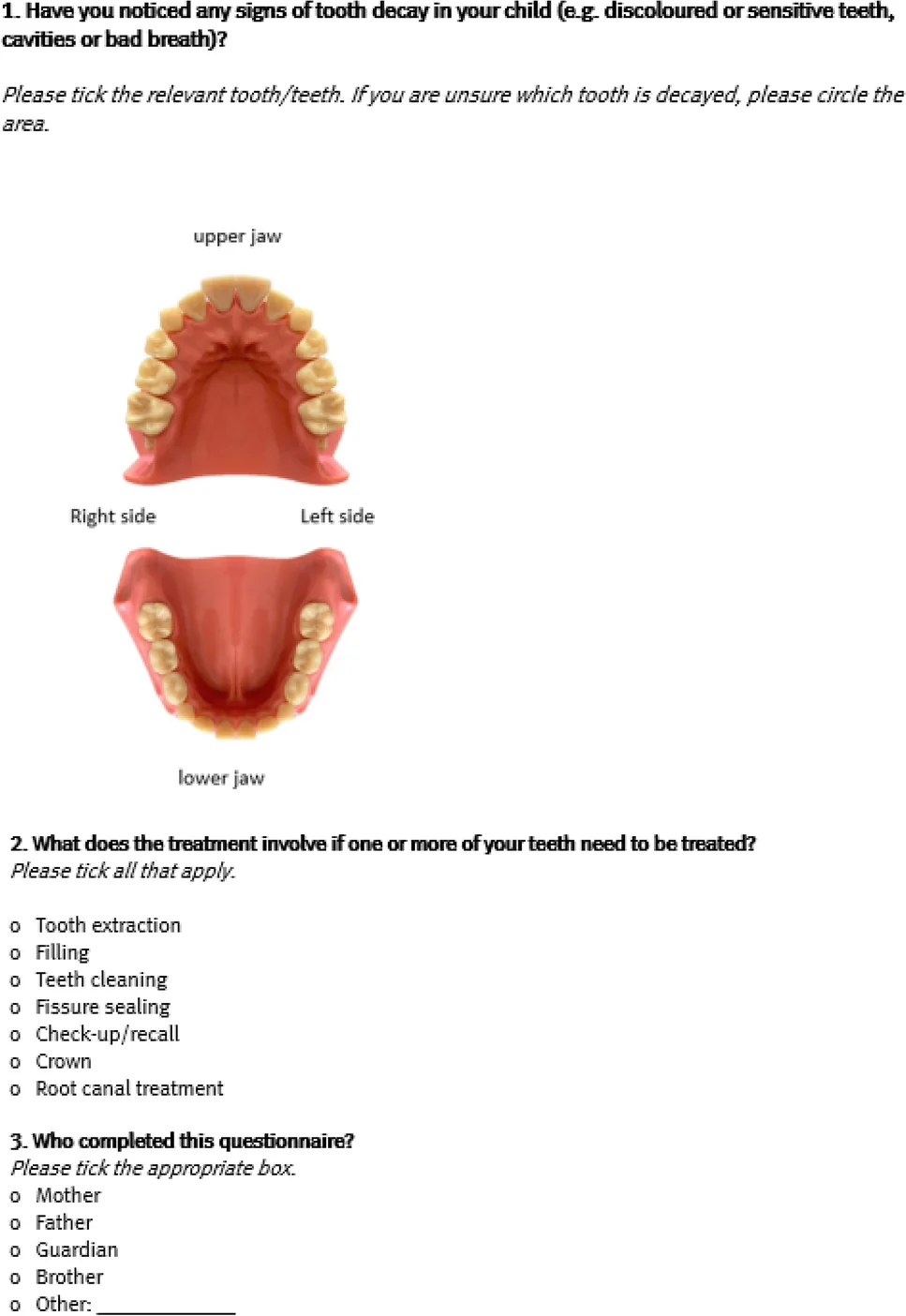
Questionnaire for accompanying adults of children (aged 6-11)

To assess comprehension, children (Fig. 1) and caregivers (Fig. 2) answered three identical questions regarding the oral health findings (presence of plaque, missing teeth, treatment need). Each of these questions was answered separately for the upper and lower jaw, resulting in six individual responses per person. In addition, caregivers answered three further questions on general understanding and therapeutic expectations (Fig. 2), resulting in a total of nine possible responses for caregivers. Responses were evaluated based on correspondence with clinical findings. A response was classified as: (1) correct—when all relevant findings were correctly identified and assigned to the appropriate teeth; (2) partially correct—when findings were recognised but incorrectly localised (e.g. caries noted but the wrong tooth) or (3) incorrect—when findings were completely missed or misrepresented. Overall scores were calculated for the number of correct and partially correct answers per individual

**Fig. 3:**
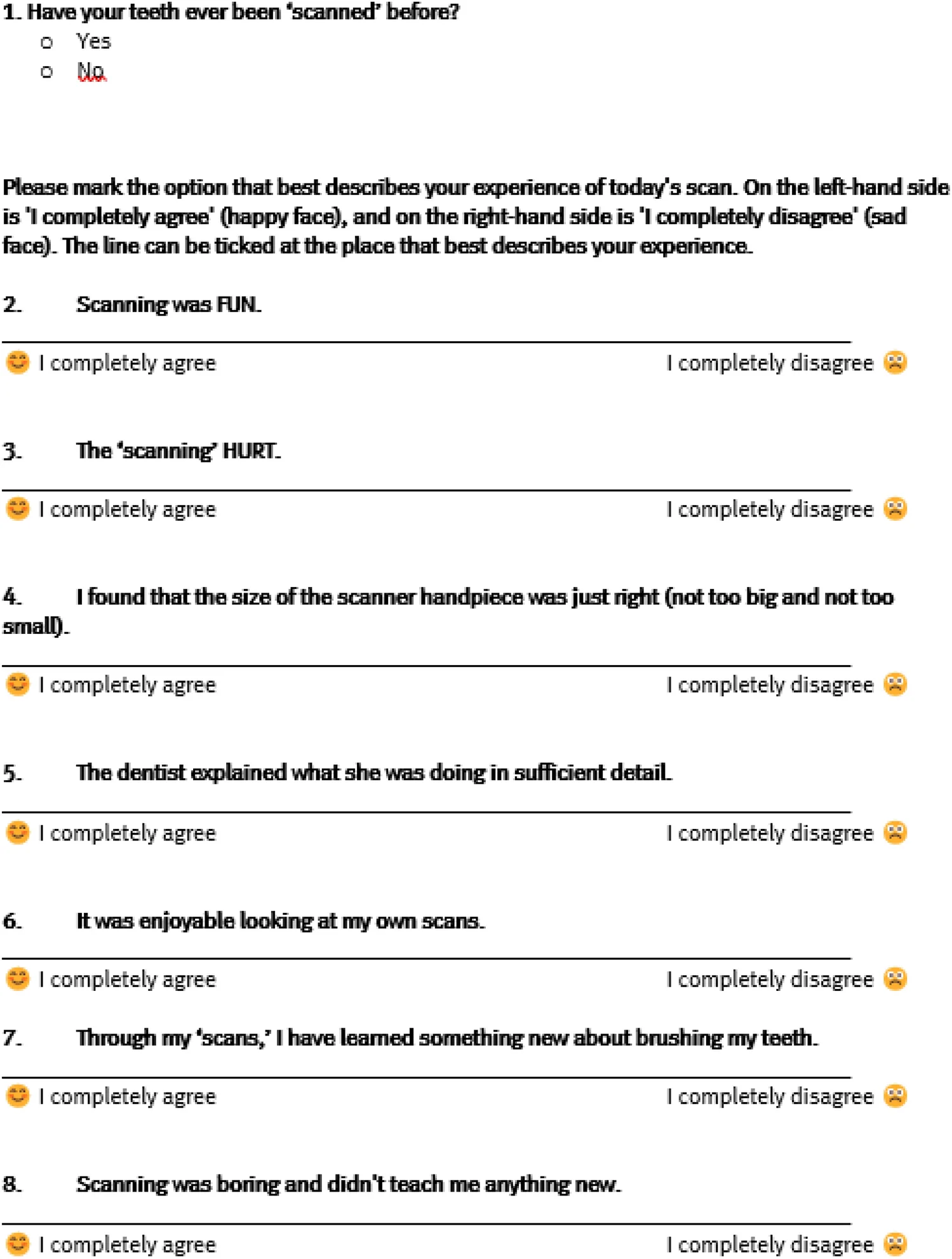
Questionnaire for children (aged 6–14) (Intervention Group)

Children in the intervention group additionally completed a visual analogue scale (VAS) questionnaire to evaluate their experience with intraoral scanning. They rated agreement with seven statements (Fig. 3) on a 100-mm line anchored with a positive response (“strongly agree”) on the left and a negative response (“strongly disagree”) on the right. The distance from the left anchor was recorded, resulting in scores between 0 and 100%.

### Statistical methods

Sample size calculations were based on a previous study (Schulz-Weidner et al. 2024a, b). The sample size was calculated with a power of 0.8, resulting in seven members per group. By adding a standard deviation of 1.1 and accounting for study dropouts or incomplete questionnaires, the group size was increased to ten. Whilst not powered to detect small differences between all subgroups, this pilot study allows for the identification of relevant trends and the feasibility of the approach. The power calculations were performed by a statistician. The final data randomisation was also conducted.

All analyses were conducted using IBM SPSS Statistics (version 29, IBM Corp., NY, USA).

Descriptive statistics (mean, standard deviation, minimum, maximum) were computed for all outcome variables. Group differences in comprehension scores (number of correct answers) between the control and intervention groups were examined using independent-samples *t *tests, separately for children and caregivers. Subgroup analysis by age group (6 to 8, 9 to 11, 12 to 14) was also performed. Effect sizes were reported as Cohen’s d.

Children in the intervention group additionally completed seven items rated on a VAS. VAS data were summarised descriptively by age group. To assess potential differences between age groups, Kruskal–Wallis tests were used. All statistical tests were two-sided, and a significance threshold of *p* < 0.05 was used.

## Results

**Fig. 4:**
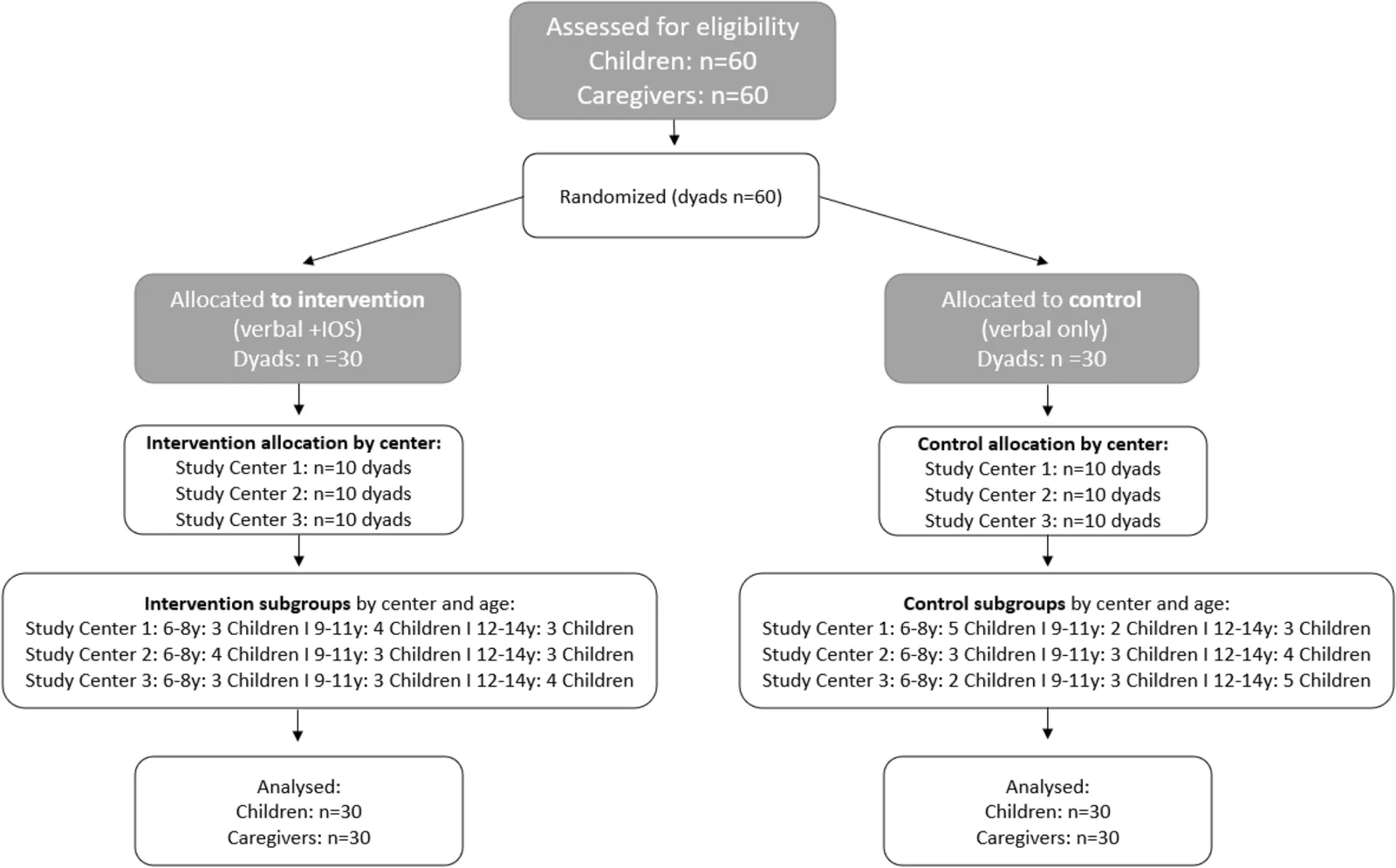
CONSORT flow chart illustrating participants’ enrolment, randomisation, group allocation, and analysis

All 120 participants completed the study and were included in the analysis. The sample consisted of 60 child-caregiver dyads, evenly distributed in the control and intervention groups. All included children showed at least one clinical finding such as plaque accumulation, gingivitis, caries, etc. Children were stratified into three age groups (6 to 8, 9 to 11 and 12 to 14 years) for subgroup analyses. Participant flow is shown in Fig. 4.

### Intervention vs. control group

**Fig. 5:**
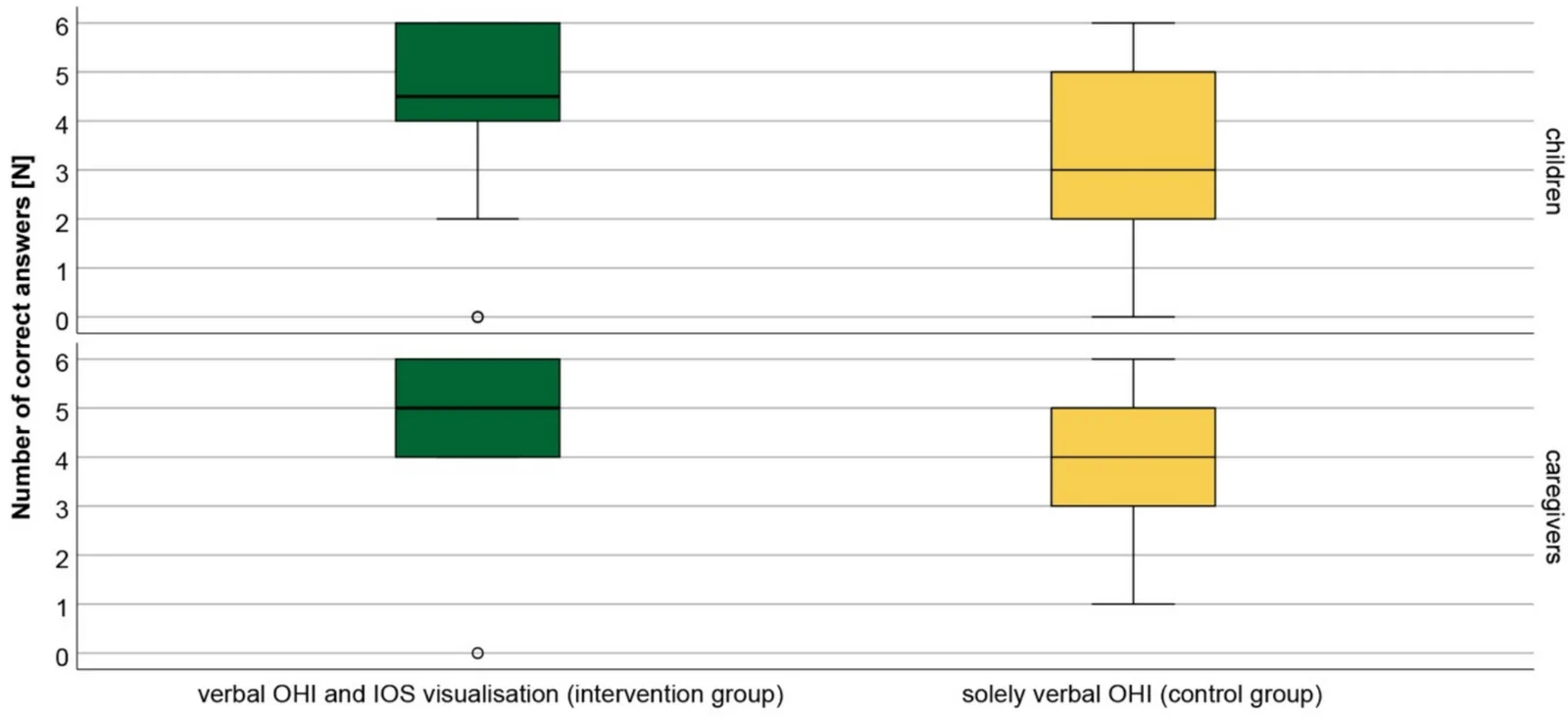
Comparison of comprehension scores (number of correct answers) for children (child) and caregivers (AP = accompanying person) between the intervention group (verbal explanation and IOS visualisation) and the control group (verbal explanation only)

Overall, both children (*n* = 30) and caregivers (*n* = 30) in the intervention group (*n* = 60) demonstrated significantly better comprehension of the dental findings than their counterparts in the control group (*n* = 60). Children in the intervention group achieved a higher mean comprehension (4.40 ± 1.61, mean ± standard deviation out of maximum six correct answers) than those in the control group (3.33 ± 1.81), with this difference reaching statistical significance (*p* = 0.019, Fig. 5). A similar pattern was observed for caregivers, whose mean score was significantly higher in the intervention group (5.03 ± 1.25) than in the control group (4.10 ± 1.37; *p* = 0.008, Fig. 5). Using the extended 9-item version of the questionnaire, caregivers in the intervention group also scored significantly higher (7.16 ± 2.14 vs. 5.35 ± 2.18; *p* = 0.013).

### Subgroup analyses: children

**Fig. 6:**
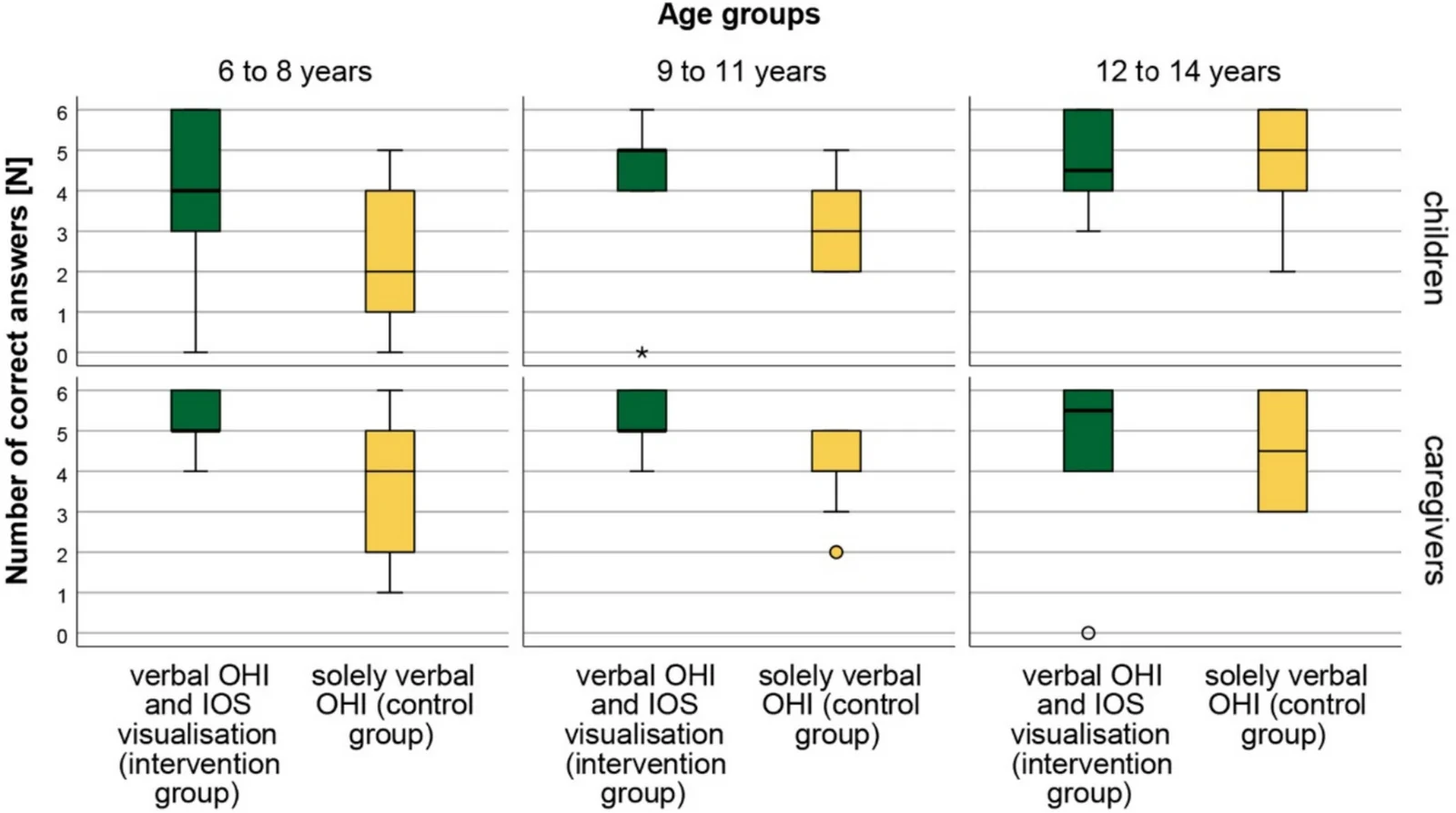
Comprehension scores of children stratified by age groups ((6 to 8 years, 9 to 11 years, 12 to 14 years) in the intervention group (verbal OHI and IOS visualisation) and the control group (solely verbal OHI)

Subgroup analysis revealed that children aged 6–8 years in the intervention group (*n* = 10) scored higher (3.90 ± 1.91) than in the control group (*n* = 10, 2.20 ± 1.75), with a large effect (Cohen’s *d* = 0.89), although this difference narrowly missed statistical significance (*p* = 0.053). Children aged 9 to 11 years scored significantly higher in the intervention group (*n* = 10, 4.50 ± 1.72) than in the control group (*n* = 10, 3.10 ± 1.20, *p* = 0.049). No statistically significant difference was observed amongst 12 to 14-year-old children (intervention group (*n* = 10), 4.80 ± 1.14; control group (*n* = 10), 4.70 ± 1.57; *p* = 0.872, Fig. 6). Within the control group (verbal OHI only), children’s comprehension scores increased numerically with age (6 to 8 years: 2.20 ± 1.75; 9 to 11 years: 3.10 ± 1.20; 12 to 14 years: 4.70 ± 1.57).

### Subgroup analyses: caregivers

Caregivers of children aged 6 to 8 years scored significantly higher in the intervention group (*n* = 10, 5.20 ± 0.79) compared to the control group (*n* = 10, 3.70 ± 1.70; *p* = 0.021). Similarly, caregivers of 9 to 11-year-olds in the intervention group achieved higher scores (5.20 ± 0.79 vs. 4.00 ± 0.94; *p* = 0.006). In the 12- to 14-year-old group, no difference was observed (intervention group: *n* = 10, 4.70 ± 1.89; control group: *n* = 10, 4.60 ± 1.35; *p* = 0.893, Fig. 6). Using the 9-item questionnaire, caregivers of 6- to 8-year-olds in the intervention group scored significantly higher than controls (7.71 ± 1.25 vs. 4.20 ± 2.59; *p* = 0.035). For caregivers of 9- to 11-years-olds, scores were also significantly higher (intervention group: 7.40 ± 0.89; control group: 5.13 ± 1.73; *p* = 0.021). No difference was found amongst caregivers of 12- to 14-year-old (intervention group: 6.43 ± 3.26; control group: 6.43 ± 2.15; *p* = 1.000). Within the control group (verbal OHI only), caregivers’ scores increased numerically with age (6 to 8 years: 3.70 ± 1.70; 9 to 11 years: 4.00 ± 0.94; 12 to 14 years: 4.60 ± 1.35). This pattern was also reflected in the 9 -item caregivers score (6 to 8 years: 4.20 ± 2.59; 9 to 11 years: 5.13 ± 1.73; 12 to 14 years: 6.43 ± 2.15)

### Intervention group: VAS questionnaire

Children in the intervention group (*n* = 30) reported a generally positive experience across all seven VAS items (Table 1). They rated the scan as fun (83.8 ± 18.9%), whilst painfulness was low (24.1 ± 33.8%). The scanning tip of the IOS was considered appropriately sized (68.7 ± 35.0%), and the explanation by the dentist was rated as clear (94.8 ± 11.3%). Watching their own scans was perceived as enjoyable (88.6 ± 20.6%), and children felt they learned something new (81.3 ± 29.5%). Low agreement was reported with the statement “it was boring and didn`t help me” (25.9 ± 36.0%). No significant age-related differences were found for the VAS items (*p* > 0.05 for all comparisons).

**Table 1:** VAS ratings (lowest score 0 to highest score 100%) by age group in the intervention group where verbal OHI was supported by the use of IOS. The short name for the respective item is mentioned in the VAS item column

VAS Item	6–8 years (mean ±SD) [%] *N*=10	9–11 years (mean ±SD) [%] *N*=10	12–14 years (mean ±SD) [%] *N*=10
Fun	79.1 ± 22.1	88.3 ± 14.8	84.1 ± 19.8
Painful	38.8 ± 43.7	25.4 ± 31.8	8.1 ± 15.4
Appropriately size (scanning tip)	67.03 ± 38.9	59.4 ± 41.3	79.6 ± 22.6
Dentist’s explanation	96.4 ± 7.7	98.7 ± 2.5	89.2 ± 17.0
Enjoyable to watch	88.7 ± 19.6	92.3 ± 16.8	84.7 ± 26.0
Informative	76.1 ± 37.4	90.8 ± 17.3	76.9 ± 31.0
Boring	25.5 ± 38.7	26.4 ± 36.4	25.8 ± 36.8

*VAS* visual analogue scale, *Mean* mean value, *SD* standard deviation

## Discussion

This multicentre cross-sectional pilot study investigated whether the integration of IOS into routine paediatric dental consultations increases oral health competence amongst children and their caregivers. The results indicate that integrating IOS into paediatric dental consultations significantly improves the comprehension of oral health findings in both children and their caregivers, particularly in younger age groups. These findings are in good accordance with previous research highlighting the value of visual aids in medical communication and patient education, particularly for individuals with limited health literacy (Houts et al. 2006; James et al. 2007; Horowitz & Kleinman 2008; Jafri et al. 2020).

In the present study, both children and caregivers in the intervention group achieved significantly higher comprehension scores (number of correct answers in the questionnaire) than those who received solely verbal OHI. The effect was particularly pronounced for younger school children aged 6 to 11 years and their caregivers. This is consistent with cognitive and developmental models suggesting that younger school children benefit disproportionately from concrete, visualised information compared to abstract verbal explanations (D’Angiulli et al. 2015). The 3D colour scans likely provided a relatable, personalised reference that enhanced comprehension and engagement, similar to findings in other areas of paediatric medicine and dentistry. Since this group in particular is in the mixed dentition phase and the permanent teeth that have just erupted into the oral cavity are more susceptible to caries, the significant effect of the additional use of IOS observed here is of great value for preventive care. However, it is important to consider that cognitive and verbal development in the age group 6–8 years is still ongoing. Children between 6 and 8 years often rely more on adult mediation, have shorter attention spans, and may interpret visual stimuli differently than older peers. Subtle variation in processing, interpretation, and memory retention may still exist but remain undetected by our current quantitative tools (Goswami & Bryant 2012).

Interestingly, no differences in comprehension were observed in the 12- to 14-year-old group. One possible explanation is that older children already have a more developed cognitive framework and dental vocabulary, allowing them to extract relevant information from the verbal explanation alone. Alternatively, the additional visual input from the IOS may have been perceived as redundant or less novel by this age group. Caregivers of older children also showed improved comprehension, mirroring the pattern observed amongst the children themselves. Several factors may contribute to this trend. One possible explanation is that older children and their caregivers may have accumulated more prior dental experience and may communicate more effectively about oral health topics. This is in line with the descriptive trend observed in the control group, where comprehension scores increased numerically with age. This observation aligns with the social cognitive framework proposed by (Bandura 2001), which emphasises the dynamic interplay between personal, behavioural, and environmental factors in shaping learning and understanding. In this context, caregivers may benefit from children’s greater verbal abilities and engagement, which can enhance joint comprehension through observational and interactive learning.

High acceptance of IOS-supported communication was indicated by VAS ratings—children rated the procedure as fun, informative, and enjoyable. Notably, the scanning procedure was not perceived as boring, and the dentists’ explanation was rated as clear. However, the VAS scores for perceived pain revealed individual variability, especially amongst the youngest children. Although the overall perception was predominantly positive, the 6- to 8-year-old group showed a mean pain rating of 38.8% ± 43.7, and the 9- to 11-year-olds reported 25.4 ± 31.8. Possible explanations include lower levels of previous dental experience, higher dental anxiety, or heightened sensitivity to new stimuli (as the IOS scanning tip) in younger age groups. Despite a visible trend toward decreasing discomfort ratings “painful” with increasing age, this effect was not statistically significant. This highlights the need to recognise individual differences in sensory perception. One plausible contributor to higher pain ratings in the youngest age group is the bulk of the scanner tip relative to oral cavity size. In this study, we used the standard Trios 4 tip; paediatric-specific tips were not available for this scanner. However, these results support previous findings on the acceptability and motivational potential of IOS in clinical settings (Schlenz et al. 2022; Schulz-Weidner et al. 2024a, b).

Future studies with larger samples and stratified individual data are necessary to verify these observations. Furthermore, a future questionnaire may include items on willingness to repeat IOS.

From a clinical perspective, the findings underscore the potential of IOS not only as diagnostic and restorative tools, but also as communication aids. The technology allows clinicians to bridge the “language gap” often encountered in paediatric dentistry by providing concrete, individualised representations of dental findings, which may be particularly valuable for children with low oral health literacy or in multilingual settings (Vanagas et al. 2009). In addition, all techniques that engage children and their caregivers in taking an interest in the child’s oral health and its promotion are valuable from a life course perspective.

The present study has several strengths, including its multicentre design, randomisation stratified by site and inclusion of both child and caregiver perspectives. However, limitations should be noted. As participants were all regular dental attendees, previous familiarity with dental settings may have increased their acceptance of intraoral scanning; however, none of the children had prior IOS experience. To ensure full understanding of the explanations and questionnaire content, we included only participants fluent in the local language. The sample size, although adequate for a pilot study, limits the generalisability of the findings. Future studies should include validated multilingual tools to increase inclusivity. Potential differences between cultural contexts (Germany vs. Finland) were not analysed separately.

Whilst the study was designed to explore age-related trends in comprehension, it is acknowledged that the broad developmental range between 6 and 12 years differs. Cognitive and linguistic abilities develop rapidly during this period, and younger children (6 to 8 years) may naturally have more difficulty in understanding oral health findings, regardless of the communication method. Although we stratified the analysis by age subgroups (6 to 8, 9 to 11, 12 to 14 years), the observed differences highlight the need for more developmentally tailored communication strategies in paediatric dentistry. Future studies could also investigate whether the number or nature of intraoral findings (e.g., plaque vs. multiple caries lesions) affects children’s ability to correctly identify and interpret their own oral health conditions.

Despite these limitations, the study provides compelling initial evidence that IOS-assisted consultations can improve understanding and experience in paediatric dental care. As this was a cross-sectional pilot study, long-term behavioural changes were not assessed. Future studies with larger, more diverse samples and validated outcome instruments are warranted. In addition, long-term outcomes such as adherence to oral hygiene instructions and recall behaviour should be examined.

## Conclusion

The use of IOS in paediatric dentistry appears to be a promising approach to improve comprehension, engagement, and communication—especially in younger school children and their caregivers. The technology is well accepted by children and offers a valuable addition to traditional verbal explanations. Its implementation in routine clinical practice and preventive programmes should be further encouraged and evaluated.

## Supplementary Information

Below is the link to the electronic supplementary material.

Supplementary file1 (DOCX 871 KB)

## Data Availability

No datasets were generated or analysed during the current study.
