# Supplementary material for: Improving oral health communication in paediatric dentistry using intraoral scanners: a multicentre pilot study
Source: Eur Arch Paediatr Dent. 2026 Apr 29;27(5):1413–24. doi: 10.1007/s40368-026-01212-z (PMC13593785; doi:10.1007/s40368-026-01212-z)
Supplement: Supplementary file 1 [file 40368_2026_1212_MOESM1_ESM.docx]

**Fig. 1S Example of an intraoral scan used during patient consultation. This visualization was used to support communication and comprehension of oral health findings, including:**

- Composite restorations on teeth 11 and 21, the restoration on 21 shows a distal defect (composite fracture),
- A previously root canal-treated primary molar (tooth 64),
- Enamel anomalies on primary molars 55, 65, 75, and 85,
- Mild enamel irregularities on permanent molars 16, 26, 36 and 46,
- Signs of localized gingivitis in anterior maxilla and mandible
